# T-box transcription factor Brachyury expression is correlated with epithelial-mesenchymal transition and lymph node metastasis in oral squamous cell carcinoma

**DOI:** 10.3892/ijo.2012.1673

**Published:** 2012-10-17

**Authors:** IKUMI IMAJYO, TSUYOSHI SUGIURA, YOSUKE KOBAYASHI, MIYUKI SHIMODA, KOTARO ISHII, NAONARI AKIMOTO, NAOYA YOSHIHAMA, IEYOSHI KOBAYASHI, YOSHIHIDE MORI

**Affiliations:** 1 Division of Maxillofacial Diagnostic and Surgical Sciences, Department of Oral and Maxillofacial Surgery; 2 Division of Maxillofacial Diagnostic and Surgical Sciences, Department of Oral Pathology, Graduate School of Dental Science, Kyushu University, Higashi-ku, Fukuoka 812-8582, Japan

**Keywords:** epithelial-mesenchymal transition, Brachyury, squamous cell carcinoma, metastasis, lymph nodes

## Abstract

The prognosis of patients with oral squamous cell carcinoma (SCC) is influenced by the presence of lymph node metastasis. Epithelial-mesenchymal transition (EMT), a process that involves events that convert adherent epithelial cells into individual migratory cells that can invade the extracellular matrix, is critical for cancer progression. Recently, the T-box transcription factor Brachyury was reported to promote EMT in human carcinoma cell lines. We analyzed the relationship between EMT (assessed by staining for E-cadherin and Vimentin) and the expression of Brachyury in association with lymph node metastasis in oral SCC. Oral SCC biopsy specimens (152 cases) were examined immunohistochemically for the expression of E-cadherin, Vimentin and Brachyury. Expression of Brachyury was correlated with EMT (p=0.035) and was significantly associated with lymph node and distant metastasis (p<0.05). Logistic regression analysis showed that Brachyury and EMT were predictive factors for lymph node metastasis (odds ratio 4.390 and 5.936, respectively) and that EMT was a predictive factor for distant metastases (odds ratio 11.786). Our findings present clinical evidence for an important role of Brachyury in EMT in oral SCC, and suggest that Brachyury and EMT patterns are useful prognostic markers.

## Introduction

Squamous cell carcinoma (SCC) is the most common malignant tumor of the oral cavity and head and neck. Oral SCC involves lymphatic metastasis, but not blood-borne metastasis, and it metastasizes to the regional lymph nodes in 30–40% of cases. Patient prognosis depends on the presence of lymph node metastasis. However, little is known about the molecular mechanisms underlying lymph node metastasis in SCC of the oral cavity. We have previously reported the relationship between lymphangiogenesis and the expression of VEGF-C and VEGF-D in association with lymph node metastasis in oral SCC.

Epithelial-mesenchymal transition (EMT) refers to a series of events that results in conversion of epithelial cells which adhere each other to individual migratory cells that can invade into extracellular matrix (1). EMT is crucial for appropriate development in the early embryo, and this process also plays an important role in adults during wound healing, tissue regeneration, and cancer progression (2). It is closely related to poor prognosis of various types of cancer [gastric (3), colon (4), breast (5,6), esophageal (7), lung (8), and ovarian (9)]. In oral cancer, EMT in tongue squamous cell carcinoma (SCC) has been reported to be related to tumor satellite formation (10) and lymph node metastasis (11). These reports suggest that EMT is a precursor of SCC metastasis and that it induces tumor cell dissemination from the primary tumor site.

The EMT events during tumor progression are controlled by genes that are normally expressed in the early embryo (e.g., *Twist, Snail, Slug, Goosecoid* and *SIP1*) (7,12–15). The transcription factors encoded by these genes can induce characteristics of mesenchymal cells, such as cell motility and invasiveness, in tumor cells. For example, the expression of Twist is increased in various types of malignant tumors, including breast cancer, prostate cancer, gastric cancer, and melanoma (16,17).

Recently, the T-box transcription factor Brachyury, a gene required for mesoderm formation during early development (18,19), was reported to promote EMT in human carcinoma cell lines (20). This study also showed that ectopic overexpression of Brachyury by gene transfection in human carcinoma cells induced characteristic changes of EMT. Based on these findings, it is proposed that EMT in cancer cells is controlled by mechanisms similar to EMT in human developmental processes.

In this study, we investigated the patterns and levels of expression of T-box transcription factor Brachyury, using immunohistochemistry on oral SCC specimens, and analyzed the relationship between Brachyury expression and EMT. We also statistically analyzed the correlation between Brachyury expression and prognosis, focusing on lymph node metastasis.

## Patients and methods

### Patients and tumor specimens

This study was based on a retrospective cohort of 152 patients who had been diagnosed with primary oral SCC and treated at the Department of Oral and Maxillofacial Surgery, Kyushu University Hospital, Fukuoka, Japan, between 1993 and 2006. All biopsies were obtained from the patients before any treatment was administered. The clinicopathological information associated with each case, including age, gender, tumor size, nodal status, location, treatment, and the presence or absence of recurrence and metastasis, was obtained from patient files. Ninety-seven men and 55 women were involved; median age was 61 years, range 24–85. All patients were staged according to the UICC TNM Classification of Malignant Tumors (21). When cervical metastasis was clinically equivocal, neck dissection was performed, and the metastases were diagnosed histologically. The protocol for the research project was approved by the appropriate Ethics Committee of Kyushu University.

### Immunohistochemistry

Biopsy samples were fixed in 10% neutralized buffered-formalin. Consecutive 4-*μ*m sections were cut and deparaffinized with xylene, rehydrated in a graded alcohol series, and heat treated with Target Retrieval Solution (Dako, Carpinteria, CA, USA) before histopathological and immunohistochemical analysis. The grade of tumor differentiation was determined using the criteria proposed by the World Health Organization (22). The histological mode of invasion was classified according to the Anneroth classification (23).

To block endogenous peroxide activity, 3% H_2_O_2_ was applied, and non-specific antibody binding was blocked with 10% normal serum in Tris-HCl buffer. The sections were incubated overnight at 4°C with the following primary antibodies: rabbit polyclonal antibody against human Brachyury (H-210, Santa Cruz Biotechnology, Santa Cruz, CA, USA); mouse monoclonal antibody against human E-cadherin (610181, BD Bioscience, San Jose, CA, USA); and goat polyclonal antibody against human Vimentin (C-20, Santa Cruz Biotechnology, Santa Cruz, CA, USA). Immunostaining was performed with the Histofine SAB-PO kit (Nichirei, Tokyo, Japan), according to the manufacturer’s instructions. The immunolocalization of the protein was visualized using a DAB substrate kit (Nichirei). Sections were counterstained with 0.5% hematoxylin, dehydrated, cleared, and mounted. Negative control staining involved substituting non-immune goat serum for the primary antibodies.

**Figure 1:**
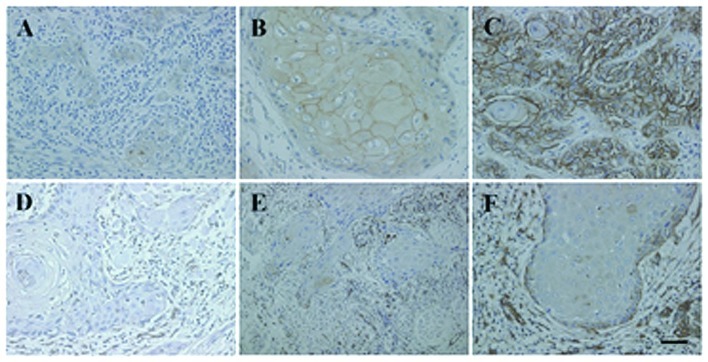
Immunohistochemical analysis of E-cadherin and Vimentin expression in SCC tissue. For the histopathological and immunohistochemical analysis, 4-*μ*m-thick sections were used as described in Patients and methods. Staining intensity of E-cadherin and Vimentin in SCC cells was classified as follows: ++, strong staining; +, moderate staining; −, negative staining as described in Table I. Photomicrographs show representative tissue staining patterns of each group. (A–C) E-cadherin; (D–F) Vimentin; (A and D) (−); (B and E) (+); (C and F) (++). Bar, 50 *μ*m.

**Figure 2:**
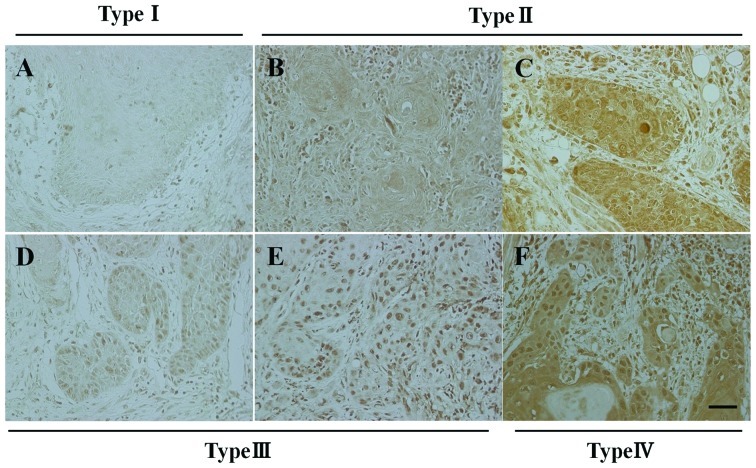
Immunohistochemical analysis of Brachyury expression in SCC tissue. Staining intensity of Brachyury in SCC cells was classified as follows: ++, strong staining; +, moderate staining; and −, negative staining as described in Table I. Pattern of cellular localization was scored as: Type I, negative staining; Type II, positive only in the cytoplasm; Type III, positive only in the nucleus; Type IV, positive in both the cytoplasm and nucleus. Photomicrographs show representative staining patterns of each group. (A) Negative (−); (B) Weak positive in the cytoplasm (+); (C) Strong positive in the cytoplasm (++); (D) Weak positive in the nucleus (+); (E) Strong positive in the nucleus (++); and (F) Strong positive in the cytoplasm and nucleus (++). Bar, 50 *μ*m.

We evaluated the staining pattern of the indicated proteins at the invasive edge of the primary tumors for all the specimens. The density of staining for Brachyury and E-cadherin was evaluated by measuring the difference between the mean density (pixels) of 10 randomly selected positive-staining fields in SCC and the mean density of background staining, using the analyze tool in Photoshop CS5 Extended (Adobe Systems Inc., San Jose, CA, USA). To avoid any measurement errors derived from staining heterogeneity, we also measured the mean density of staining for each protein in normal cells (lymphocytes for Brachyury and normal epithelium for E-cadherin) as positive controls in the same sample, and the ratio of staining density between SCC and positive control was calculated. This ratio was taken to represent the expression level of the protein. For Vimentin, the number of the positive SCC cells in a low-power field was counted. The results were classified into 3 groups for each protein as shown in Table I: ++, strong staining; +, moderate staining; and −, negative (Figs. 1 and 2). Two independent observers blinded to the patient status scored the samples.

### Immunofluorescence staining

Sections were cut and mounted as described above and heat treated with Target Retrieval Solution (Dako) before immunofluorescence staining. Non-specific antibody binding was blocked with 3% BSA in phosphate-buffered saline (PBS) for 1 h at room temperature. The sections were incubated overnight at 4°C with primary antibodies as described above. Following three 10-min washes in PBS, sections were incubated with secondary antibodies for 1 h at room temperature. The secondary antibodies (Invitrogen, Carlsbad, CA, USA) used were: for Brachyury, Alexa Fluor 498 goat anti-mouse, catalog no. A11034; for E-cadherin, Alexa Fluor 350 goat anti-mouse, catalog no. A11029; for Vimentin, Alexa Fluor 594 donkey anti-goat, catalog no. A11058. After additional washes, images were captured using a Z-axis-controlled microscope with a CCD camera (BZ-8000; Keyence, Osaka, Japan) and processed for deconvoluted fluorescence imaging.

### Statistical analysis

Statistical analysis was performed with the statistical software package SPSS for Windows (Abacus Concepts, Berkeley, CA, USA). Relationships between staining intensity of markers and various clinicopathological factors were assessed with the χ^2^ test. To define independent risk factors for lymph node metastasis and distant metastasis, univariate and multivariate analysis were performed with a logistic regression analysis. The survival rate was estimated with the Kaplan-Meier method and analyzed using the log-rank test. Differences were considered significant at p<0.05.

## Results

### EMT pattern in oral SCC

We examined EMT in oral SCC lesions by assessing loss of E-cadherin and gain of Vimentin using immunohistochemistry. E-cadherin was localized on the cell membrane of SCC cells but was lost in some cases, whereas Vimentin was stained in the cytoplasm of SCC cells and in the stromal tissue (Fig. 1). We categorized E-cadherin and Vimentin staining by staining intensity and scored the staining patterns as EMT (E-cadherin-negative, Vimentin-positive) or not EMT.

Table II summarizes clinicopathological data of all cases and E-cadherin and Vimentin immunoreactivity. The relationship between E-cadherin and Vimentin expression and clinicopathological factors was analyzed with the χ^2^ test. The staining intensity of E-cadherin on SCC cells was significantly associated with lymph node involvement (p= 0.049), distant metastasis (p= 0.001), and tumor differentiation (p= 0.041), while the intensity of Vimentin staining was associated with lymph node involvement (p= 0.009) and distant metastasis (p= 0.002). The relationship between EMT pattern and clinicopathological factors was also analyzed with the χ^2^ test. Lymph node involvement (p=0.009) and distant metastasis (p=0.001) were significantly correlated with presence of the EMT pattern.

### Pattern and level of expression of Brachyury in oral SCC

We next examined the expression of Brachyury in oral SCC lesions using immunohistochemistry. Brachyury protein was localized in the cytoplasm and/or the nucleus of SCC cells. Brachyury was occasionally detected in lymphocytes around the SCC nests and in stromal cells surrounding the SCC nests. SCC cells in the invasive front tended to be stained strongly (Fig. 2). We categorized Brachyury staining by staining intensity in the cytoplasm or nucleus and by pattern of cellular localization: Type I, negative staining; Type II, positive only in the cytoplasm; Type III, positive only in the nucleus; and Type IV, positive in both the cytoplasm and nucleus. The relationship between Brachyury expression (intensity and pattern) and clinicopathological factors was analyzed with the χ^2^ test (Table III).

The rate of positive expression of Brachyury was 71.0% (Type I, 28.9%; Type II, 11.8%; Type III, 27.0%; and Type IV, 32.2%). Nuclear staining intensity of Brachyury in SCC cells was significantly associated with clinical T stage (p=0.001), lymph node involvement (p= 0.003), tumor differentiation (p=0.043), and the pattern of invasion (p=0.024), while cytoplasmic intensity of Brachyury was associated only with lymph node involvement (p=0.028) and the pattern of invasion (p=0.022).

The staining pattern of Brachyury on SCC cells was more significantly associated with clinicopathological factors than intensity of Brachyury; the pattern was associated with clinical T stage (p=0.001), lymph node involvement (p=0.004), tumor differentiation (p= 0.03), and the pattern of invasion (p= 0.006).

### Molecular localization of Brachyury and EMT markers in oral SCC

**Figure 3:**
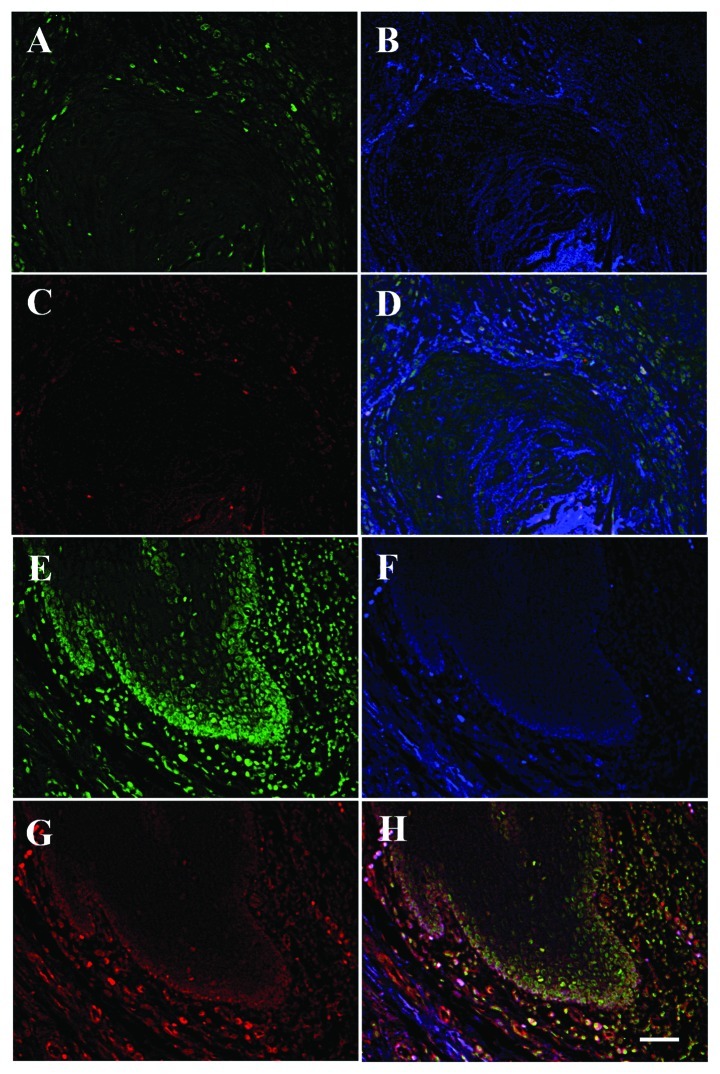
Triple immunofluorescence staining analysis of Brachyury, E-cadherin, and Vimentin expression in SCC tissue. Each protein was visualized by secondary antibodies: Brachyury, green; E-cadherin, blue; and Vimentin, red. (A–D) Representative staining of EMT-negative, Brachyury-negative SCC. (E–H) Representative staining of EMT-positive, Brachyury-positive SCC. (A and E) Brachyury; (B and F) E-cadherin; (C and G) Vimentin; and (D and H) Merged image. Bar, 50 *μ*m.

To find direct evidence that Brachyury is associated with EMT, we analyzed localization of Brachyury, E-cadherin, and Vimentin in oral SCC tissues by triple immunofluorescence staining. Fig. 3A shows that Brachyury-negative cells strongly express E-cadherin. In contrast, stromal cells express Brachyury and Vimentin. Fig. 3B shows that SCC cells expressing both Brachyury and Vimentin were found at the invasive front of the primary nest.

### Relationships between Brachyury expression and EMT in oral SCC

The relationship between Brachyury expression pattern and EMT (staining intensity of E-cadherin and Vimentin or scored pattern) was analyzed with the χ^2^ test (Table IV).

Brachyury expression pattern was significantly correlated with Vimentin expression (p=0.002). In particular, Type IV staining was strongly associated with expression of Vimentin. Brachyury expression pattern was also significantly correlated with EMT (p=0.035).

### Logistic regression analysis of the predictive factors for lymph node and distant metastasis

To examine the significance of the predictive factors for lymph node metastasis, a logistic regression analysis was performed (Table V). A univariate analysis showed that EMT, positive expression of Brachyury, and Vimentin, and negative expression of E-cadherin were related to lymph node metastasis (odds ratios 5.936, 4.39, 3.368, and 0.444, respectively), while EMT, lacking expression of E-cadherin, and Vimentin were related to distant metastasis (odds ratios 11.768, 0.113, and 4.558, respectively). Multivariate analysis also showed that positive expression of Brachyury was related to lymph node metastasis (odds ratio 3.952), while negative expression of E-cadherin was related to distant metastasis (odds ratio 0.141). Brachyury expression exhibited the highest odds ratios (4.390 and 3.952) among the predictive factors for lymph node metastasis, and E-cadherin exhibited the most significant odds ratios (0.113 and 0.141) among the predictive factors for distant metastasis.

### Correlation between Brachyury expression and/or EMT and survival time

**Figure 4:**
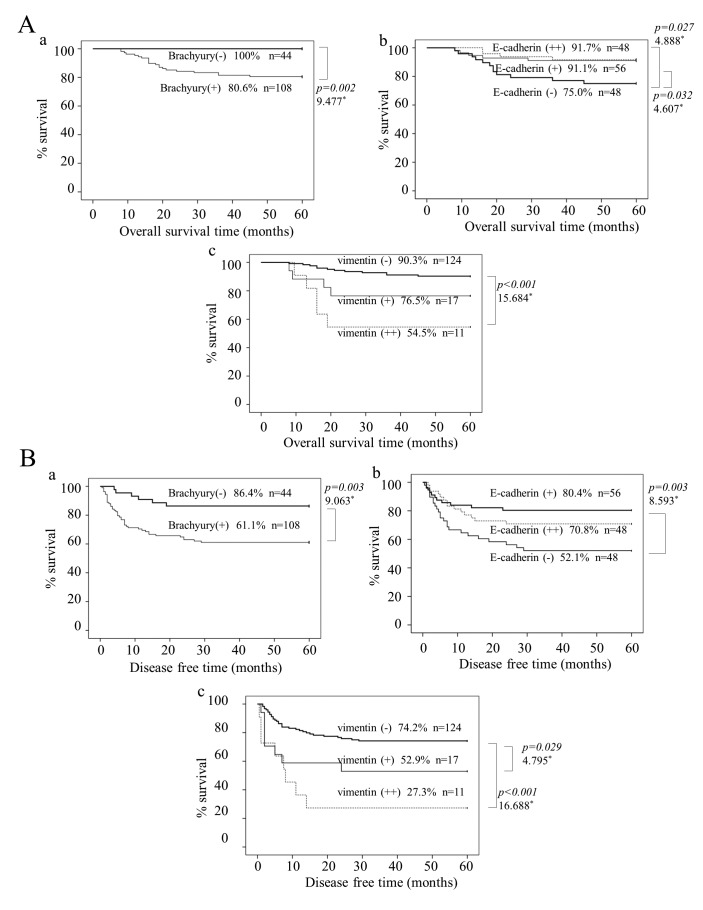
Kaplan-Meier survival analysis. Survival rate was estimated with the Kaplan-Meier method and analyzed using the log-rank test. Overall (A) and disease-free (B) survival of patients with tumors of the indicated status for (a) Brachyury, (b) E-cadherin, and (c) Vimentin are shown. ^*^χ^2^ statistics.

To investigate whether Brachyury expression and/or EMT pattern in biopsy specimens predicts outcome in patients with oral SCC, Kaplan-Meier analysis for staining intensity of Brachyury, E-cadherin, and Vimentin of the overall (Fig. 4A) and disease-free (Fig. 4B) survival times was performed (Fig. 4). The 5-year overall survival rate of patients with Brachyury-positive SCC (80.6%) was significantly less than that of the negative counterpart (100%, log-rank test, p= 0.002; χ^2^, 9.477). The survival time of patients with E-cadherin-negative SCC (−) was significantly less than that of the weak (+) and strong (++) positive counterparts (log-rank test, p= 0.032; χ^2^ 4.607; p= 0.027; χ^2^, 4.888, respectively). The survival time of patients with strong Vimentin-positive (++) SCC was significantly less than that of the negative counterpart (log-rank test, p<0.001; χ^2^, 15.684).

**Figure 5:**
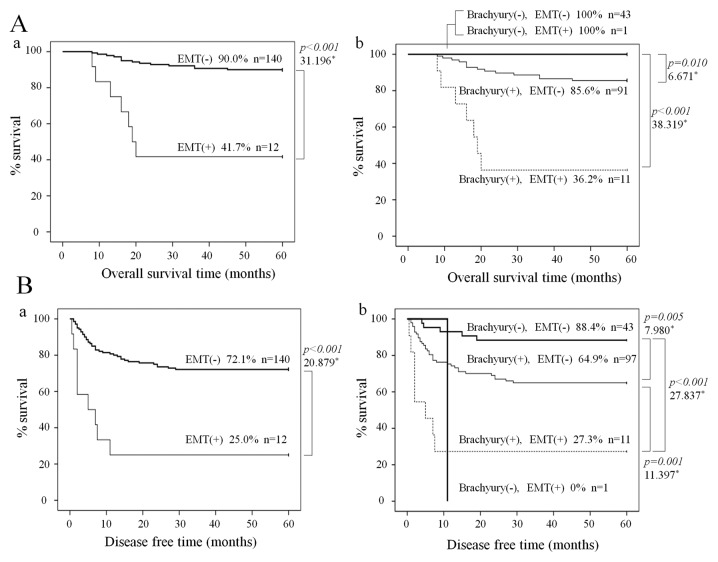
Kaplan-Meier survival analysis for EMT pattern and Brachyury expression. Survival rate was estimated with the Kaplan-Meier method and analyzed using the log-rank test. Overall (A) and disease-free (B) survival of patients with tumors of the indicated status for (a) EMT and (b) Brachyury with EMT are shown. ^*^χ^2^ statistics.

We also analyzed the survival time for EMT pattern or Brachyury expression with EMT pattern by the Kaplan-Meier method (Fig. 5A, overall survival rate; Fig. 5B, disease-free survival rate). The survival time of patients with SCC exhibiting the EMT pattern was strikingly shorter than in EMT-negative groups. The 5-year overall survival rate in these patients was 41.7% (log-rank test, p= 0.001; χ^2^, 31.196) and the 5-year disease-free survival rate was 25.0% (log-rank test, p<0.001; χ^2^, 20.879). Moreover, survival time of patients with Brachyury expression and EMT pattern was significantly shorter than in Brachyury-negative groups; 5-year overall survival rate was 36.2% (log-rank test, p= 0.01; χ^2^, 38.319) and 5-year disease-free survival rate was 27.3% (log-rank test, p<0.001; χ^2^, 27.837).

## Discussion

Cancer invasion and metastasis are crucial events in disease progression and largely determine prognosis of cancer patients. Many factors regulate cancer cell behavior, and correlation between clinical prognoses and expression of these factors in various tumors has led to their use as prognostic markers. Recently, EMT has been identified as not a factor but a crucial event in cancer invasion and metastasis during which many factors are simultaneously regulated (24,25). In this regard, EMT patterns in tumor tissue are likely to be useful for estimating cancer prognosis.

We used loss of E-cadherin and gain of Vimentin as a marker of EMT, and we found a significant correlation between EMT pattern and lymph node involvement or distant metastasis (Table II). Interestingly, Vimentin expression showed a stronger relationship with distant metastasis than did E-cadherin expression. Loss of E-cadherin is thought to be a hallmark of EMT, because loss of E-cadherin loosens cell-cell adhesion enabling cancer cells to escape from the primary cancer nest (26). For example, in one recent study, the downregulation of E-cadherin shows significant difference compared to the pattern of invasion, tumor satellite formation, and tumor satellite size (10). On the other hand, Vimentin overexpression in cancer tissues also correlates with tumor growth, tumor invasion, and poor clinical outcome (3,27). In oral cancer, expression of Vimentin significantly increases in groups stratified by tumor satellite distance (10). Vimentin overexpression induces tyrosine kinase expression and results in cancer cell migration (28). This could explain the strong correlation between Vimentin expression and distant metastasis.

The mechanisms by which EMT is regulated in cancer cells are still not well understood. Recently, Fernando *et al*(20) reported that Brachyury, a gene required for mesoderm formation during early development, promotes EMT in human carcinoma cell lines. Their study showed that overexpression of Brachyury in human cancer cells induced characteristic changes of EMT, including elevated level of mesenchymal markers, decrease of epithelial markers, and increased cell migration and invasion. In our immunohistochemical analysis, Brachyury expressing cancer cells lost E-cadherin and gained Vimentin expression, consistent with previous reports and Fig. 3. Based on these data, we hypothesized that Brachyury expression is linked to EMT and correlated with disease prognosis. Therefore, we analyzed the relationship between Brachyury expression and clinicopathological findings (Table III). Brachyury expression correlated with clinical T stage, lymph node involvement, tumor differentiation, and pattern of invasion. Importantly, cellular localization of Brachyury is more important factor than staining intensity. Brachyury is a T-box transcription factor that transduces cellular signals by translocation to the nucleus. Nuclear localization of Brachyury was significantly related to malignant phenotypes in oral cancer (Table III). A role for Brachyury in promotion of EMT is supported by our finding that Brachyury cellular localization was significantly correlated with Vimentin expression and EMT pattern (Table IV).

It is also noteworthy that EMT and Brachyury expression in oral SCC showed a significant correlation with lymph node involvement (Table V). These findings suggest that Brachyury is one of the key elements in the control of EMT and that EMT is one of the most important events in the development of malignant phenotypes.

The existence of self-renewing, stem-like cells within tumors, called cancer stem cells (CSCs), has been proved. CSCs form a minor population in cancer cells within a tumor and are defined by their ability to establish new tumors. Hence, CSCs are also called ‘tumor-initiating cells’ (29). During cancer cell metastasis, which is often enabled by EMT (30), cancer cells which escape from primary site would require a self-renewal capability similar to that exhibited by stem cells in order to generate a new tumor in distant site. In this regard, the EMT process may also give a self-renewal capability to metastatic cancer cells. Indeed, emerging evidence of a direct interaction between EMT and CSCs has been reported (20,31,32). Expression of SOX2, a member of the SOX (SRY-related high mobility group box) family, was recently shown to be significantly associated with poor prognosis in oral tongue SCC (33). SOX2 was originally characterized as an important regulator of the maintenance of embryonic stem cell pluripotency (34). It is possible that SOX2 can function to maintain CSCs.

Like SOX2, Brachyury is an important gene in embryonic development and induces EMT in early embryonic stages and in cancer cells (35). These similarities raise the possibility that Brachyury is also an important regulator of CSC maintenance. Notably, recent clinical evidence suggests that Brachyury regulates CSCs in colorectal cancer (36). The report suggests that Brachyury regulates Nanog in mesenchymal-like cancer cells to impose a plastic state, allowing competence of cells to respond to signals prompting invasion or metastasis. In preliminary work in our laboratory, Brachyury knockdown by short hairpin RNA in oral cancer stem cells completely inhibited the EMT phenotype and cancer stem cell phenotypes (tumorigenicity and sphere formation) *in vitro* (Sugiura, unpublished data). If Brachyury directly regulates CSCs, Brachyury expression could be a predictor of the effects of chemotherapy and radiotherapy, because CSCs were shown to be resistant to chemotherapy and radiotherapy *in vitro*(37,38). Furthermore, Brachyury could be a therapeutic target for anti-CSC therapy. Therefore, the effects of Brachyury in CSCs should be further investigated.

In conclusion, this study presents clinical evidence for an important role for Brachyury in EMT in oral SCC, and suggests that Brachyury expression and EMT patterns are useful prognostic markers.

## Figures and Tables

**Table I:** Classification of staining density.

		−	+	++	Unit
Brachyury	Nuclear	<0.2	0.2–0.7	>0.7	
Control: lymphocyte	Cytoplasm	<0.1	0.1–0.25	>0.25	Ratio
E-cadherin		<0.37	0.37–0.54	>0.54	
Control: normal epithelium					
Vimentin		0	0–10	>10	Cells/field
No. of positive cells in a ×400 field					

$$\text{Expression}\;\text{strength}=\frac{\left(\text{mean}\;\text{density}\;\text{of}\;\text{positive}\;\text{signal}\;)\;-\;(\text{mean}\;\text{density}\;\text{of}\;\text{background}\;\text{staining}\right)}{\left(\text{mean}\;\text{density}\;\text{of}\;\text{positive}\;\text{control})\;-\;(\text{mean}\;\text{density}\;\text{of}\;\text{back}\;\text{ground}\;\text{staining}\right)}$$

**Table II:** Relation between EMT and clinicopathological factors.

	Cases	E-cadherin				Vimentin				EMT		
		−	+	++	p-value	−	+	++	p-value	−	+	p-value
Age												
≤65	88	30	28	30	NS	76	8	4	NS	83	5	NS
>65	64	18	28	18		48	9	7		57	7	
Sex												
Male	97	29	33	35	NS	78	12	7	NS	90	7	NS
Female	55	19	23	13		46	5	4		50	5	
Clinical stage												
T1	37	13	13	11	NS	33	3	1	NS	36	1	NS
T2	62	17	25	20		50	8	4		58	4	
T3	29	12	9	8		25	3	1		25	4	
T4	24	6	9	9		16	3	5		21	3	
Tumor site												
Buccal mucosa	14	7	5	2	NS	10	3	1	0.021	13	1	NS
Upper gingiva	7	2	4	1		3	1	3		5	2	
Lower gingiva	39	10	13	16		34	3	2		36	3	
Tongue	81	27	26	28		67	10	4		75	6	
Oral floor	11	2	8	1		10	0	1		11	0	
Lymph node involvement												
Positive	56	24	15	17	0.049	39	9	8	0.009	47	9	0.009
Negative	96	24	41	31		85	8	3		93	3	
Distant metastases												
Positive	13	10	0	3	0.001	7	2	4	0.002	8	5	0.001
Negative	139	38	56	45		117	15	7		132	7	
Tumor differentiation												
Well	132	37	50	45	0.041	107	15	10	NS	123	9	NS
Moderate	19	11	6	2		16	2	1		16	3	
Poor	1	0	0	1		1	0	0		1	0	
Pattern of invasion												
1	2	1	1	0	NS	2	0	0	0.030	2	0	NS
2	37	10	14	13		32	3	2		35	2	
3	82	23	31	28		69	5	8		77	5	
4	31	14	10	7		21	9	1		26	5	

χ^2^ test: NS, not significant. p<0.05.

**Table III:** Relation between expression pattern of Brachyury and clinicopathological factors.

	Cases	Nuclear				Cytoplasm				Expression pattern				
		−	+	++	p-value	−	+	++	p-value	I	II	III	IV	p-value
Age														
≤65	88	34	23	31	NS	55	16	17	NS	29	5	26	28	0.039
>65	64	28	20	16		30	15	19		15	13	15	21	
Sex														
Male	97	36	25	36	NS	47	22	28	0.041	22	14	25	36	NS
Female	55	26	18	11		38	9	8		22	4	16	13	
Clinical stage														
T1	37	27	2	8	<0.001	24	4	9	NS	21	6	3	7	0.001
T2	62	25	23	14		35	11	16		16	9	19	18	
T3	29	5	11	13		15	6	8		4	1	11	13	
T4	24	5	7	12		11	10	3		3	2	8	11	
Tumor site														
Buccal mucosa	14	6	5	3	NS	9	1	4	NS	4	2	5	3	NS
Upper gingiva	7	4	2	1		2	3	2		2	2	0	3	
Lower gingiva	39	11	13	15		20	11	8		6	5	14	14	
Tongue	81	36	22	23		47	13	21		28	8	19	26	
Oral floor	11	5	1	5		7	3	1		4	1	3	3	
Lymph node involvement														
Positive	56	13	22	21	0.003	26	10	20	0.028	7	6	19	24	0.004
Negative	96	49	21	26		59	21	16		37	12	22	25	
Distant metastases														
Positive	13	4	3	6	NS	5	2	6	NS	2	2	3	6	NS
Negative	139	58	40	41		80	29	30		42	16	38	43	
Tumor differentiation														
Well	132	58	37	37	0.043	74	27	31	NS	43	16	31	42	0.030
Moderate	19	3	6	10		10	4	5		0	2	10	7	
Poor	1	1	0	0		1	0	0		1	0	0	0	
Pattern of invasion														
1	2	1	0	1	0.024	2	0	0	0.022	1	0	1	0	0.006
2	37	21	8	8		20	9	8		14	7	6	10	
3	82	35	25	22		54	13	15		28	7	26	21	
4	31	5	10	16		9	9	13		1	4	8	18	

χ^2^ test: NS, not significant. Significant, p<0.05.

**Table IV:** Relationships among Brachyury expression, EMT markers, and EMT.

Brachyury expression pattern	E-cadherin				Vimentin				EMT		
	−	+	++	p-value	−	+	++	p-value	Negative	Positive	p-value
I	14	16	14	NS	42	1	1	0.002	43	1	0.035
II	4	8	6		11	4	3		16	2	
III	15	18	8		38	2	1		40	1	
IV	15	14	20		33	10	6		41	8	

χ^2^ test: NS, not significant. p<0.05.

**Table V:** Logistic regression analysis of predictive factors.

	Univariate analysis			Multivariate analysis		
	Odds ratio	p-value	95% CI	Odds ratio	p-value	95% CI
Lymph node involvement						
Brachyury						
Negative vs. positive	4.39	0.001	1.799–10.714	3.952	0.040	1.563–9.988
E-cadherin						
Negative vs. positive	0.444	0.024	0.220–0.897	0.478	0.089	0.204–1.120
Vimentin						
Negative vs. positive	3.368	0.005	1.443–7.864	2.020	0.215	0.665–6.131
EMT						
Negative vs. positive	5.936	0.010	1.534–22.965	1.533	0.653	0.238–9.890
Distant metastases						
Brachyury						
Negative vs. positive	2.381	0.272	0.506–11.215	1.836	0.475	0.347–9.713
E-cadherin						
Negative vs. positive	0.113	0.001	0.029–0.432	0.141	0.023	0.026–0.766
Vimentin						
Negative vs. positive	4.558	0.012	1.398–14.860	2.506	0.468	0.210–29.969
EMT						
Negative vs. positive	11.786	<0.001	3.051–45.527	1.533	0.771	0.086–27.307
